# Prediction of the Sound Absorption Coefficient of Three-Layer Aluminum Foam by Hybrid Neural Network Optimization Algorithm

**DOI:** 10.3390/ma15238608

**Published:** 2022-12-02

**Authors:** Han Mi, Wenlong Guo, Lisi Liang, Hongyue Ma, Ziheng Zhang, Yanli Gao, Linbo Li

**Affiliations:** 1 College of Metallurgical Engineering, Xi’an University of Architecture and Technology, Xi’an 710055, China; 2 Shaanxi Metallurgical Engineering Technology Research Center, Xi’an 710055, China; 3 Xinjiang Key Laboratory of Aluminum-Based Electronic and Electrical Materials, Wulumuqi 830012, China

**Keywords:** open-cell aluminum foam, composite structure, sound absorption, generalized regression neural network, equilibrium optimization algorithm

## Abstract

The combination of multilayer aluminum foam can have high sound absorption coefficients (SAC) at low and medium frequencies, and predicting its absorption coefficient can help the optimal structural design. In this study, a hybrid EO-GRNN model was proposed for predicting the sound absorption coefficient of the three-layer composite structure of the aluminum foam. The generalized regression neural network (GRNN) model was used to predict the sound absorption coefficient of three-layer composite structural aluminum foam due to its outstanding nonlinear problem-handling capability. An equilibrium optimization (EO) algorithm was used to determine the parameters in the neuronal network. The prediction results show that this method has good accuracy and high precision. The calculation result shows that this proposed hybrid model outperforms the single GRNN model, the GRNN model optimized by PSO (PSO-GRNN), and the GRNN model optimized by FOA(FOA-GRNN). The prediction results are expressed in terms of root mean square error (RMSE), absolute error, and relative error, and this method performs well with an average RMSE of only 0.011.

## 1. Introduction

Noise pollution is one of the four major environmental problems in the world at present; long-term exposure to a noisy environment affects people’s everyday life and rest, reducing people’s quality of life [1], and causes more serious can induce cardiovascular and cerebrovascular diseases [2,3]. In recent years, sound-absorbing materials have been widely used. Traditional sound-absorbing materials such as sound-absorbing felt, sound-absorbing cotton, and rock wool, have a short service life, severe environmental pollution, and other problems. Open-cell aluminum foam, a new type of porous acoustic material [4], has low specific gravity, non-combustibility, corrosion resistance, and easy processing while absorbing noise [5,6]. There is no secondary pollution during production, use, and end-of-life recycling, and it is called “green material” [7]. The available research results show that the open-cell aluminum foam composite structure with double-layer sandwich cavity has better sound absorption performance at low and medium frequencies [8,9,10,11,12,13], and this study tries to investigate the sound absorption coefficient of the three-layer aluminum foam plus composite cavity structure at low and medium frequencies [14]. The absorption coefficient measurement of three-layer aluminum foam requires experiments through standing wave tubes. Testing the absorption coefficient of the three-layer plus cavity open-cell aluminum foam composite structure is complicated and challenging. In this case, an accurate model for sound absorption estimation is necessary [15,16,17].

The transfer matrix method is traditionally used to obtain the absorption coefficients of multilayer acoustic materials [17,18]. The commonly used models are the D-B empirical model [19], the equivalent fluid model proposed by Johnson-Champoux-Allard [20], and the Miki model obtained by Miki by modifying the D-B model [21]. The absorption coefficients calculated by several of the above models and the transfer matrix method have some accuracy. However, the accuracy for calculating the low-frequency case is low and requires unique structural parameters (flow resistivity, characteristic length, and curvature coefficient). With the development of intelligent algorithms and neural networks, more and more researchers are choosing neural networks to predict the acoustic properties of materials. Artificial neural networks (ANN) [22,23,24,25,26,27], radial basis function neural networks (RBF) [9], and generalized regression neural networks (GRNN) [28], are commonly used to predict sound absorption coefficients. 

The above neural network prediction methods have good accuracy but require a large amount of training data and are unsuitable for predicting small sample data. Among them, GRNN, as a powerful regression tool with a dynamic network structure, has nonlinear solid mapping capability, fault tolerance, and robustness [29,30]. However, the disadvantage is that its prediction accuracy is often affected by the spread value. A good set of spread values can significantly improve the accuracy of GRNN. Optimization algorithms can be used to optimize the spread value of GRNN. Afshin Faramarzi et al. (2019) proposed the equilibrium optimization algorithm (EO) [29,30], whose principle is inspired by the control volume mass balance, which can estimate the kinetics and equilibrium state, where each particle and its concentration as a search agent will randomly update the current best concentration value. The optimum solution we need is the concentration value obtained when the system reaches equilibrium. In addition to the more novel EO algorithm, researchers have also used the particle swarm optimization algorithm (PSO-GRNN) [31] and the fruit fly optimization algorithm (FOA-GRNN) [32,33] in different neural networks for optimization adjustments to suit different research contents.

In this study, the simple structural parameters of aluminum foam are used as input variables to construct a GRNN model with several parameters and sound absorption coefficients. The spread values in the model are optimized to obtain a model suitable for small sample sizes and have high accuracy. The error between predicted and experimental values is used to measure model accuracy. The study’s key is to optimize the spread value. The GRNN model with suitable spread value can guide the design of material structure parameters according to the model and obtain a more efficient sound absorption structure.

In this study, Section 2 introduces the principle of the GRNN model and the specific steps of combining the EO algorithm. Section 3 is the acquisition of the structural parameters of aluminum foam in the experiment and the testing of the sound absorption coefficient, and proposes the criteria for measuring the model. Section 4 is the discussion of the model prediction results and experimental results, and Section 5 is the summary of the content of this study.

## 2. Equilibrium Optimizer-Generalized Regression Neural Network

### 2.1. Generalized Regression Neural Network

**Figure 1:**
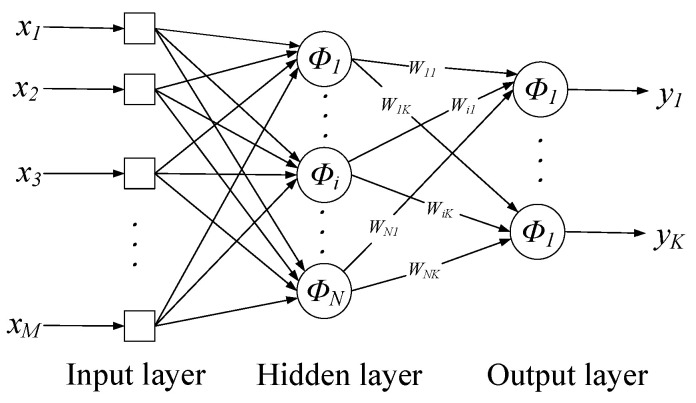
Schematic diagram of the GRNN architecture.

Donald F. Specht introduced the Generalized Regression Neural Network in 1991, a radial basis neural network. The GRNN has strong nonlinear mapping capability, flexible network structure, high fault tolerance, and robustness, which is suitable for solving nonlinear problems. The GRNN is established based on non-parametric regression [34,35]. The GRNN usually constructs its input pattern representation through an intermediate layer that provides a specific number of units. Each unit of this intermediate layer is intentionally defined and characterized by an activation function. The GRNN is a kind of neural network whose function is to construct the internal representation of the input model through the proper configuration of the middle layer and can personalize the “features” of each input belonging to the training set [36]. The GRNN is a four-layer forward network, as shown in Figure 1 [37]: the first layer is the input layer, and the number of neurons is equal to the dimension of the input vector; the second layer is the pattern layer, and the number of neurons is equal to the number of learning samples; the third layer is the summation layer, sum over all the neurons in the hidden layer; the fourth layer is the output layer, the number of nodes is equal to the dimension of the output vector [30].

With the sample data as the posterior condition, Parzen non-parametric estimation is performed [38], and the network output is calculated according to the principle of maximum probability. Assume that *x* and *y* are two random variables whose joint probability density is *f*(*x, y*). If the observed value of *x* is known to be *x*_0_, the regression of *y* for *x* is shown in Equation (1):

(1) $$E\left(y|x_{0}\right)=y\left(x_{0}\right)=\frac{\int_{-\infty }^{0}yf\left(x_{0},y\right)dy}{\int_{-\infty }^{0}f\left(x_{0},y\right)dy}$$

*y*(*x*_0_) is the predicted output of *y* with an input of *x*_0_. Using Parzen non-parametric estimation, the density function *f*(*x*_0_, *y*) can be estimated from the sample data set ${\left\{x_{i},y_{i}\right\}}_{i=1}^{n}$ according to Equation (2):

(2) $$f\left(x_{0},y\right)=\frac{1}{n{\left(2\pi \right)}^{\frac{p+1}{2}}\sigma^{p+1}}\sum_{i=1}^{n}\exp\left[-d\left(x_{0},x_{i}\right)\right]\exp\left[-d\left(y,y_{i}\right)\right]$$

where $d\left(x_{0},x_{i}\right)={\sum_{j=1}^{p}\left[\left(x_{0j}-x_{ij}\right)/\sigma \right]}^{2},d\left(y,y_{i}\right)={\left[y-y_{i}\right]}^{2}$*n* is the sample size, *P* is the dimension of the random variable *x*, and σ is the spread value. Substitute Equation (2) into Equation (1), and the result is shown in Equation (3):

(3) $$y\left(x_{0}\right)=\frac{\sum_{i=1}^{n}\left\{\exp\left[-d\left(x_{0},x_{i}\right)\right]\int_{-\infty }^{+\infty }\mathrm{yexp}\left[-d\left(y,y_{i}\right)\right]dy\right\}}{\sum_{i=1}^{n}\left\{\exp\left[-d\left(x_{0},x_{i}\right)\right]\int_{-\infty }^{+\infty }\exp\left[-d\left(y,y_{i}\right)\right]dy\right\}}$$

Simplification Equation (3), and the result is shown in Equation (4):

(4) $$y\left(x_{0}\right)=\frac{\sum_{i=1}^{n}y_{i}\exp\left[-d\left(x_{0},x_{i}\right)\right]}{\sum_{i=1}^{n}\exp\left[-d\left(x_{0},x_{i}\right)\right]}$$

It can be seen from Equation (4) that the spread value is crucial to the prediction effect of the GRNN. When the spread value is tremendous, the predicted value is approximately the mean value of all sample dependent variables. On the contrary, when the spread value tends to 0, the predicted value is very close to the value of the training sample. However, the predicted effect deteriorates sharply once the new input is given. Therefore, obtaining the appropriate spread value is necessary for different input samples to obtain good prediction results. This paper intends to use the Equilibrium Optimizer Algorithm to obtain the spread value suitable for different input samples so that the optimized GRNN can still obtain accurately predicted values even with a small amount of data and achieve a low prediction error.

### 2.2. Equilibrium Optimizer Algorithm

The Equilibrium Optimization Algorithm adopted in this paper is inspired by control volume mass balance models that estimate both dynamic and equilibrium states [30]. The mass balance equation reflects the process of mass entry, departure, and generation in the control volume. It is generally described by a first-order differential equation, as shown in Equation (5):

(5) $$V\frac{dC}{dt}=Q_{eq}-QC+G$$

where *V* is the control volume; *C* is the concentration in the control volume; *Q* is the volumetric flow rate into or out of the control volume; *C_eq_* represents the concentration inside the control volume when there is no mass generation (equilibrium state), and *G* is the mass generation rate inside the control volume.

By solving the differential equation described by Equation (5), we can find:

(6) $$C=C_{eq}+\left(C_{0}-C_{eq}\right)F+G\left(1-F\right)/\lambda V$$

(7) $$F=\exp\left(-\lambda \left(t-t_{0}\right)\right)$$

where *F* is the exponential term coefficient, *λ* is the flow rate, and *C*_0_ is the initial concentration of the control volume at time *t*_0_.

The specific operation procedure and parameters of the algorithm are designed as follows:

Step 1. The initialization of algorithm parameters, the algorithm performs random initialization within the upper and lower bounds of each optimization variable, as shown in Equation (8):

(8) $$C_{i}^{0}=C_{\min}+r_{i}\left(C_{\max}-C_{\min}\right),i=1,2,\cdots ,n$$

where *C*_min_, *C*_max_ are the lower and upper bound vectors of the optimization variables, respectively; *r_i_* represents the vector of random numbers of individual *i*, whose dimensionality is the same as that of the optimization space, and each element value is a random number from 0 to 1.

**Figure 2:**
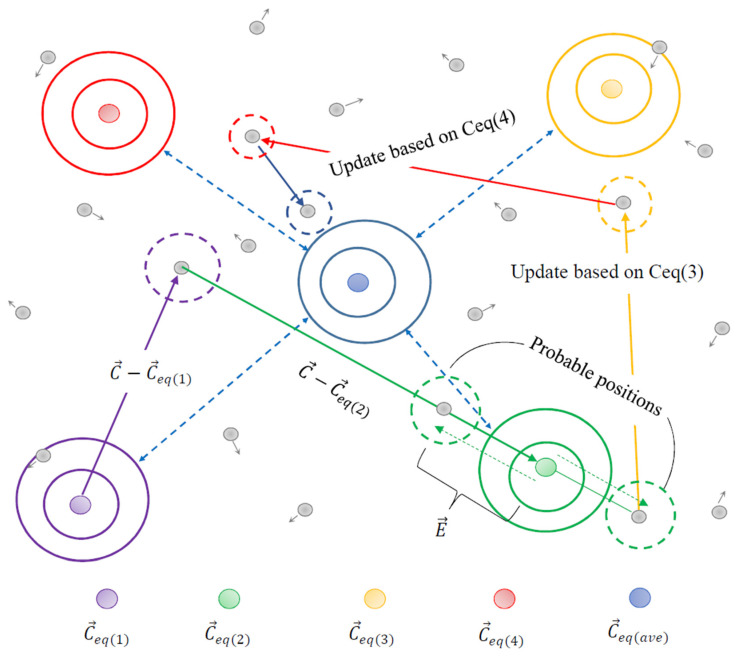
Equilibrium optimization algorithm finding optimal solution process.

Step 2. Determine the current balance pool status to improve the global search capability of the algorithm and avoid getting trapped in low-quality local optimal solutions, the optimal individual of the equilibrium state in Equation (6) will be selected from the five currently optimal candidate solutions (Figure 2) [30]. The pool of equilibrium states formed by these candidate solutions is shown in Equation (9):

(9) $$C_{eq,pool}=\left\{C_{eq,1},C_{eq,2},C_{eq,3},C_{eq,4},C_{eq,ave}\right\}$$

where *C_eq_*_,1_, *C_eq_*_,2_, *C_eq_*_,3_, *C_eq_*_,4_ are the best four solutions found as of the current iteration, respectively; *C_eq_*_, *ave*_ represents the average state of these four solutions. It is worth noting that the probability of these five candidate solutions being selected is the same, all being 0.2.

Step 3. Update the index term coefficients according to Equation (10):

(10) $$F=a_{1}sign\left(r-0.5\right){\left[e^{-\lambda t}-1\right]}_{}^{}$$

where *a*_1_ is the constant coefficient of the weight of the global search; sign is the sign function; *r*, λ both represent the vector of random numbers whose dimension is the same as the dimension of the optimization space, and each element value is a random number from 0 to 1.

Step 4. Equations (11) and (12) update the quality generation coefficients further to enhance the local search capability of the algorithm:

(11) $$G=G_{CP}\left(C_{eq}-\lambda C\right)$$

(12) $$G_{CP}=\left\{ \begin{array}{l} 0.5r_{1}\;r_{2}\geq 0.5\\ 0\;r_{2}<0.5 \end{array}\right.$$

where *G_CP_* is a vector of generation rate control parameters; *r*_1_ is a vector of random numbers whose dimension is the same as the dimension of the optimization space, and each element value is a random number from 0 to 1; *r*_2_ is a random number in the range of 0 to 1.

Step 5. The final optimal solution is obtained according to Equation (13):

(13) $$C=C_{eq}+(C-C_{eq})F+G(1-F)/\lambda V$$

### 2.3. EO-GRNN for Predicting Sound Absorption Coefficient

The sound absorption coefficient of a three-layer composite structured aluminum foam proposed in this study is predicted by the EO-GRNN, where the spread parameters of the GRNN are automatically determined by the equilibrium optimization (EO) algorithm. The concrete steps of the EO-GRNN model are as follows:

Step 1. Data preprocessing, normalizing the input data and dividing it into training and test sets, applying the training set data to the equilibrium optimization algorithm to determine the optimal spread value.

Step 2. Parameter initialization, the parameters to be determined are the number of particles, the maximum number of iterations, and the exploration coefficient a_1_, and start the loop iteration.

Step 3. Calculate the current fitness value and spread value. The error of GRNN determines the fitness function.

Step 4. The optimal concentration and fitness values are stored and updated with the number of iterations to ensure that the values for each loop iteration are exhausted. 

Step 5. Update the three important parameters of the balancing algorithm, including candidate concentration of the equilibrium pool (*C_eq_*), exponential term (*F*), and generation rate (*G*).

Step 6. Calculate the updated concentration value (spread value in the GRNN) and the fitness value.

Step 7. Determine if the maximum number of iterations is reached. If so, apply the best concentration value (spread value in the GRNN) to the GRNN and calculate the current output value (sound absorption coefficient). Otherwise, return to Step 3.

**Figure 3:**
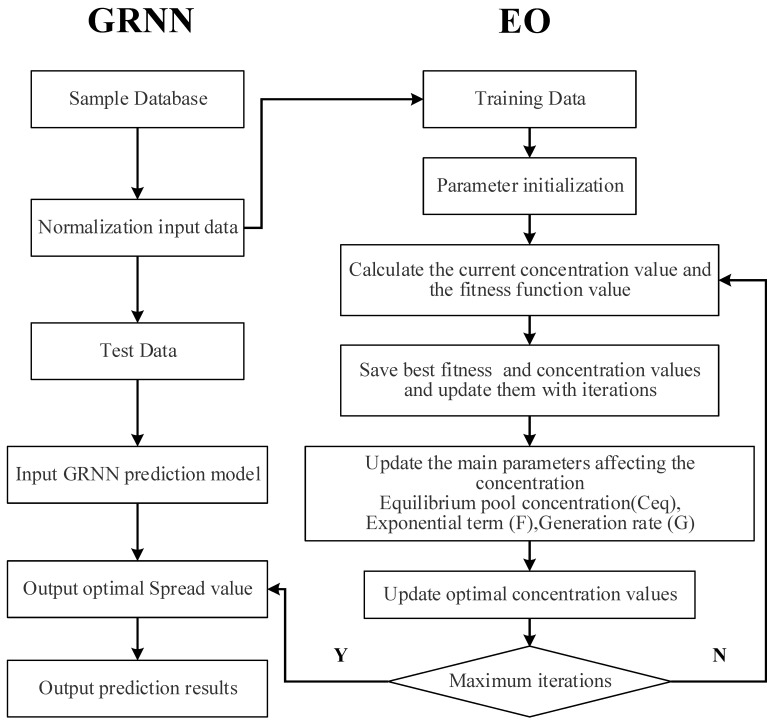
The flowchart of the EO-GRNN model.

The flowchart of the EO-GRNN model is shown in Figure 3.

## 3. Experimental

### 3.1. Experiment Data

**Figure 4:**
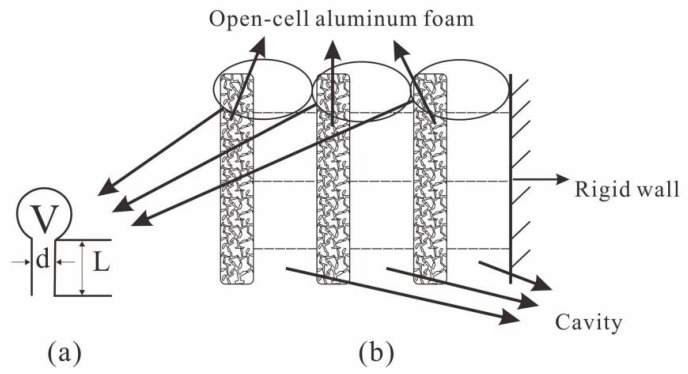
Helmholtz resonance structure. (**a**) Helmholtz single structure; (**b**) diagram of adding a back cavity.

The experimental material chosen in this paper is open-cell aluminum foam with different porosity. As a metal-based porous acoustic material, the open-cell aluminum foam conforms to the principle of acoustic absorption of porous acoustic materials. Its acoustic performance is severely limited by frequency. It cannot achieve a high absorption coefficient at low and medium frequencies. The open-cell aluminum foam post-air layer is equivalent to a Helmholtz resonator [39]. The Helmholtz resonator has the characteristics of high sound absorption in a narrower frequency band to compensate for the performance defects of single-layer aluminum foam. The three-layer aluminum foam with separate posterior air layers further improves the sound absorption performance in the target frequency band [14]. The schematic diagram is shown in Figure 4. The complex pore structure of the open-cell aluminum foam increases the inlet tortuosity (similar to the neck of a Helmholtz resonator), which makes it more effective than the resonance absorption structure of an ordinary perforated plate. The three-layer open-cell aluminum foam composite structure is similar to many Helmholtz resonators connected in parallel and then in series, with significantly higher absorption coefficients and wider absorption bands than a single layer of open-cell aluminum foam.

**Figure 5:**
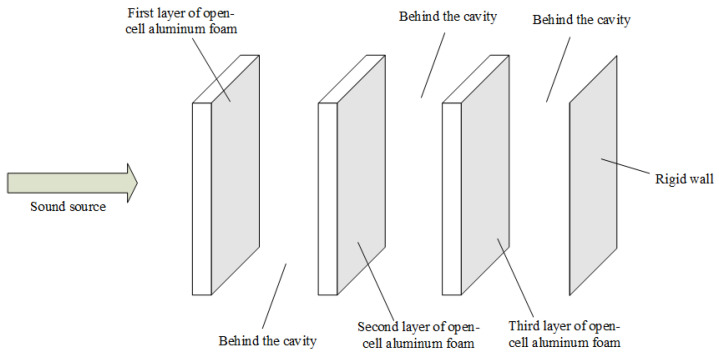
Absorption model of multilayer composite open-cell aluminum foam structure.

**Figure 6:**
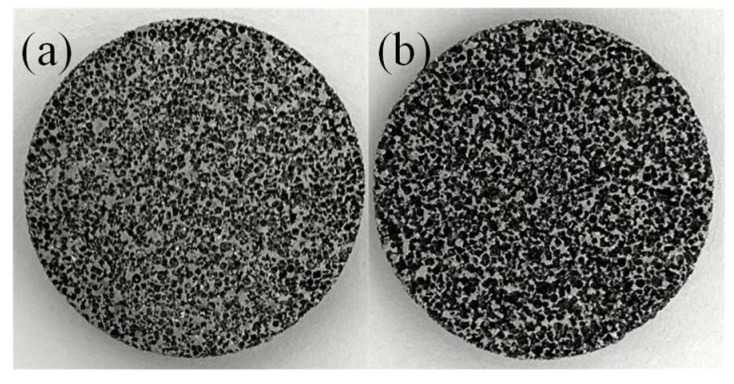
Open-cell aluminum foam: (**a**) The less dense open-cell aluminum foam; (**b**) the denser open-cell aluminum foam.

**Figure 7:**
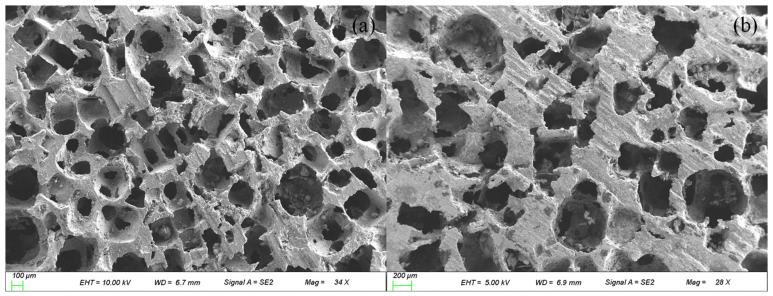
SEM images of the open-cell aluminum foam: (**a**) The less dense open-cell aluminum foam; (**b**) the denser open-cell aluminum foam.

The three-layer composite structure we designed is shown in Figure 5. In the experiment stage, we specially designed 16 open-cell aluminum foam structures using two different porosity of open-cell aluminum foam. The two materials were cut into 30 mm diameters by wire cutting. The morphologies of the two materials are shown in Figure 6, the morphology was characterized by scanning electron microscopy (SEM), and the SEM images of the two materials are shown in Figure 7.

**Figure 8:**
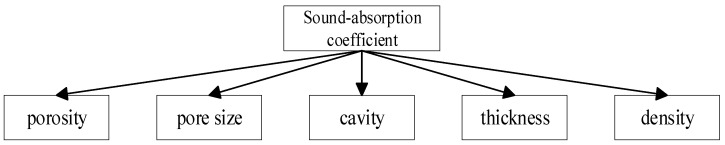
Five factors affecting the sound absorption coefficient of aluminum foam.

Among the above, two kinds of open-cell aluminum foam with different porosity have five thicknesses of 3 mm, 5 mm, 6 mm, 8 mm, and 10 mm, and they are correspondingly coded as *A*_1_*~A*_5_ and *B*_1_*~B*_5_. Then, the encoding of the composite structure can then be written in a form such as *M_a_L_i_N_a_L_i_O_a_L_i_* (*M* represents the first layer of open-cell aluminum foam, *N* represents the second layer of open-cell aluminum foam, *L* represents the third layer of open-cell aluminum foam, subscript *a* = 1, 2, 3, 4, 5. L represents the depth of the back cavity, *i* = 0, 10, 20, 30, 40, 50). The EO-GRNN was used to predict the sound absorption coefficient of the composite structure with the basic parameters of some easily measured by open-cell aluminum foam (as shown in Figure 8) as inputs.

In this paper, we take some easily measurable basic parameters such as porosity, pore size, density, thickness *d* (mm), and cavity depth *a* (mm) as inputs [40]. The foamed materials were first to cut into regular shapes. The porosity of open-cell aluminum foam was calculated by the direct mass-volume calculation method, using an analytical balance to weigh its mass M. After sealing the open-cell aluminum foam, volume V was measured by the Archimedes drainage method. Finally, the porosity can be expressed as shown in Equation (14); the pore size is measured by the bubble method, the aluminum foam specimen is immersed in water so that the specimen in the open pores to saturation, and then with compressed gas, will be the specimen pores in the impregnated liquid blow out. When the gas pressure gradually increases from small to a particular value, the gas can be immersed in the liquid from the pores to push away, and bubbles, the measurement of the first bubble when the pressure difference, can use the Laplace equation as shown in Equation (15) to find the pore size of porous materials; the thickness and diameter are measured using vernier calipers to calculate the volume of the specimen *v*, use an electronic balance to measure its mass *m* and calculate its density *ρ,* as shown in Equation (16):

(14) $$\psi =\left(1-\frac{M}{V\rho_{s}}\right)\times 100\%$$

where ψ is the porosity; *M* is the mass of the sample; *V* is the volume of the sample; $\rho_{s}$ is the density of aluminum;

(15) $$r=\frac{2\sigma \cos\theta }{\Delta p}$$

where *r* is the pore diameter, *σ* is the surface tension of the immersed liquid, *θ* is the immersion angle, and Δp is the pressure difference.

(16) $$\rho =\frac{m}{v}$$

In this paper, porosity, pore size, density, thickness and cavity depth are taken into account, as shown in Figure 8, and each parameter of the materials applied to is shown in Table 1.

### 3.2. Measurement of the Sound Absorption Coefficient

**Figure 9:**
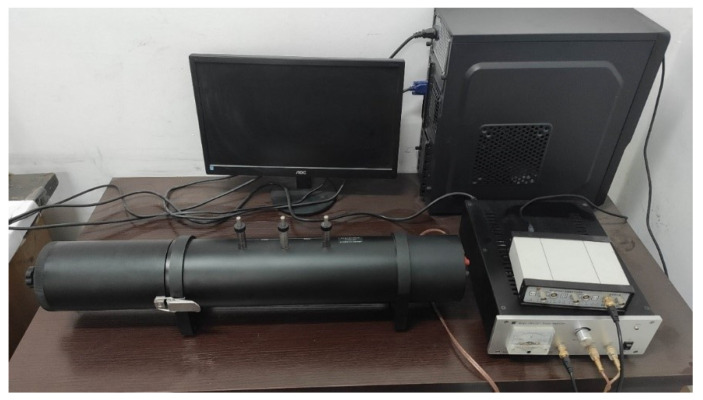
Sound absorption coefficient test equipment.

The transfer function method measured the sound absorption coefficient using the international standard ISO 10534-2:1998. Hangzhou Aihua Instruments Co. (Hangzhou, China) produced the impedance tube AWA8551 used in the experiment (Figure 9). Before measuring the sound absorption coefficients, the materials were washed by the ultrasonic wave and dried under constant temperature to ensure that no impurities affected the sound absorption capacity of the materials. The whole measurable frequency range of AWA8511 is from 20 Hz to 10,000 Hz. Since we mainly deal with medium and high-frequency bands noise in workshops, factories, and other places, we process the sample into a cylinder with a diameter of 30 mm. Then, the sound absorption coefficient of low and medium frequencies from 500 Hz to 6300 Hz is measured by a small tube. Among the various frequencies, we chose intermediate frequencies such as 500 Hz, 630 Hz, 800 Hz, 1000 Hz, 1250 Hz, 1600 Hz, and 2000 Hz to represent this measurement.

First, the sound absorption coefficients of the 10 types of open-cell aluminum foams in Table 1 were measured. The measurement results are shown in Table 2, from which we can see that the absorption coefficient of single-layer aluminum foam is low and the sound absorption performance is poor, and the absorption coefficient increases with the increase of the thickness of the open-cell aluminum foam, but the effect is not apparent. We combined the above aluminum foams to obtain 16 kinds of composite structured aluminum foams with an air layer, and their sound absorption coefficient values are shown in Table 3. The maximum average absorption coefficient increased from 0.202 to 0.954, and the absorption coefficient of each frequency band improved significantly.

### 3.3. Prediction of the Sound Absorption Coefficient

To verify the accuracy of the EO-GRNN model in predicting the sound absorption coefficient of three-layer aluminum foam composite structures, models of a single GRNN, the GRNN model optimized by the particle swarm optimization algorithm (PSO-GRNN), and the GRNN model optimized by the fruit fly optimization algorithm (FOA-GRNN), were also developed in this study for simultaneous prediction of sound absorption coefficients with the EO-GRNN. The same data were selected for each group of models.

The input data need to be preprocessed before simulation. The model’s input parameters include porosity, pore size, thickness, and density. The order of magnitude difference between the data is significant and needs to be normalized to map their range to −1 and 1. The mathematical expression for normalization is given in Equation (17), while the output data absorption coefficients measured by impedance tubes do not require further processing:

(17) $$y=\frac{y_{max}-y_{min}}{x_{max}-x_{min}}\cdot (x-x_{min})+y_{min}$$

where *y* is the normalized value, *x* is the actual value, *y_min_* = −1, *y_max_* = 1, and *x_min_* and *x_max_* are the minimal and maximal values of each input factor, respectively.

For the 16 sets of data in Section 3.2, groups 1–11 are selected as the training group for the EO-GRNN, and groups 12–16 are used as the validation group to verify the model’s accuracy. The parameters that need to be used before the simulation is the number of particles in the EO model, which represents the number of equilibrium optimizations per iteration of 30, the maximum number of iterations is 500, the exploration factor *a*_1_ is 2, the range of spread values is [0.001,50]. The initialization process obtains other model parameters. In addition, the fitness function in the EO model is the root mean square error between the predicted and experimental values. The root-mean-square error can reflect the deviation of the predicted value of each frequency in each group from the experimental value as a whole, reflecting the accuracy of the predicted value in all the tested frequencies, whose expression is shown in Equation (18):

(18) $$RMSE=\sqrt{\frac{\sum_{i=1}^{n}{\left(\alpha_{i}^{m}-\alpha_{i}^{c}\right)}^{2}}{n}}$$

where *α_i_^m^* denotes the experimentally measured absorption coefficient at each frequency, *α_i_^c^* denotes the absorption coefficient predicted by the four models at each frequency, and *n* denotes the number of frequencies, *n* = 7. The experimental environment includes the Matlab 2018b, the GRNN toolbox, and self-written MATLAB programs.

## 4. Results and Discussion

In the previous section, we introduced the calculation of RSEM as a model measure. Table 4 also presents the mean values of absolute and relative errors of the four prediction models and the spread values obtained during the optimization process. 

**Figure 10:**
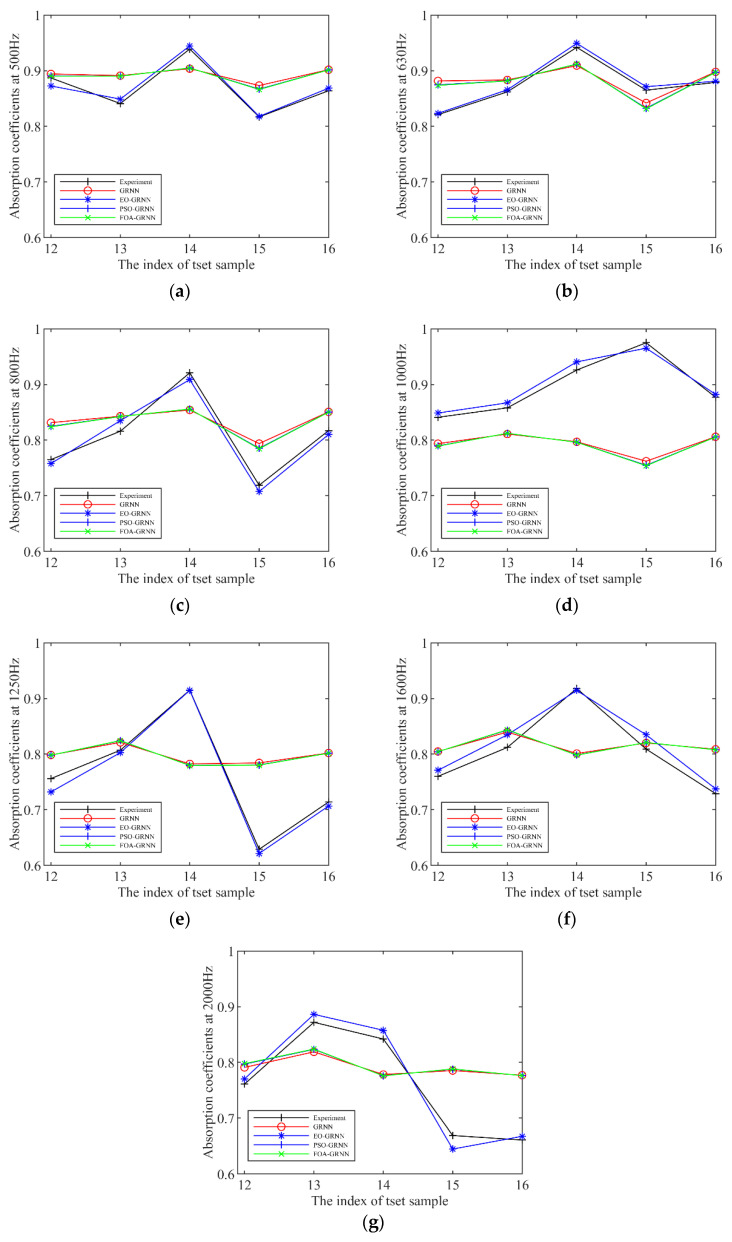
Experimental measurements and predicted values of sound absorption coefficients for groups 12–16 acoustic structures at different frequencies by four prediction models. Figure (**a**–**g**) represent the absorption coefficients of the sound-absorbing structures at 500 Hz, 630 Hz, 800 Hz, 1000 Hz, 1250 Hz, 1600 Hz, and 2000 Hz, respectively.

**Figure 11:**
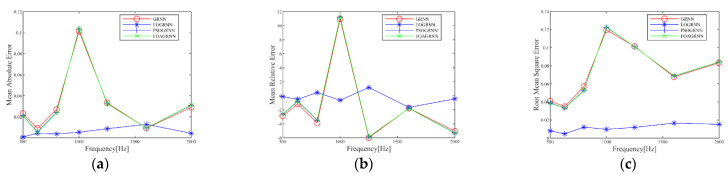
Error plots of the prediction models and experimental values of the four absorption coefficients with frequency change. (**a**) Absolute error; (**b**) relative error; and (**c**) root mean square error.

Figure 10 plots the simulated and experimental values of absorption coefficients at each frequency for the five validation groups, and the EO-GRNN has high accuracy at each frequency. The predicted values of the EO-GRNN model at each frequency in Figure 10 are closer to the experimental measurements. In contrast, the other three models are closer to the tested values only at individual frequencies (630 Hz and 1600 Hz), and the same phenomenon is shown in Figure 11. It shows the mean values of absolute error, relative error, and root-mean-square error for each model in the 12th to 16th validation groups, respectively, with the root-mean-square error less than 0.02 and the relative error less than 3%, which are within the error range of (−3%,3%) [32], indicating that the model has a more accurate performance. It can also be seen from the figure that the prediction curves of the GRNN, the PSO-GRNN, and the FOA-GRNN are relatively close to each other, mainly because the spread values obtained by these models are relatively close to each other, which leads to this phenomenon. The prediction results of each model near 1000 Hz are poor, probably because of the impaired value of 1000 Hz when the absorption coefficient is tested in the impedance tube, which leads to the poor prediction performance of several models, while the EO-GRNN still shows a high accuracy, which indicates the robustness of the model [29]. As can be seen from Table 4, the EO-GRNN performs the best, while the POS-GRNN and the FOA-GRNN have essentially the same accuracy, which can also be reflected in the absorption coefficient plot. This situation may be because PSO and FOA choose a fixed step size for exploration during optimization [41]. At the same time, EO updates the current search parameters at each iteration during optimization and compares the target values among the five candidate concentrations to avoid falling into a local optimum solution.

**Figure 12:**
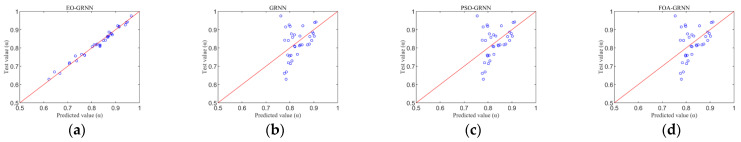
Plots of all predicted values of the four prediction models with their corresponding experimental values consisting of points: (**a**) EO-GRNN; (**b**) GRNN; (**c**) PSO-GRNN; and (**d**) FOA-GRNN.

Figure 12 shows the trend plots of the predicted and tested values of the absorption coefficients obtained under the four optimization algorithms. If the distance between each point and y = x (red line in the figure) is short and uniformly distributed around it, it indicates a better prediction [42]. In contrast, the results in the figure show that only the EO-GRNN has excellent accuracy, while the other three are less uniformly distributed and have a more considerable distance from y = x. It indicates that the overall accuracy is not enough, and the distributions of the FOA-GRNN and the PSO-GRNN are very close, indicating that they all fall into the same local optimal solution when optimizing the spread values and do not obtain the optimal global solution.

In contrast, the EO equilibrium algorithm performs a separate optimal search for four of the candidate pools due to the uniform division of the entire solution space into five candidate regions when performing the optimization of the spread values of the GRNN, and the last candidate pool is the optimal solution for the last search. During the subsequent search, more accurate values are obtained, and the algorithm itself is designed with *a*_1_, *r*, *λ* to increase the exploration capability and the homogeneity of the search process so that the parameters get more globally meaningful values in each candidate pool, ensuring the accuracy of the values in each candidate pool [30]. These unique designs are not available in the traditional PSO and FOA algorithms. They will keep advancing and searching according to their respective search trends and miss values with sure accuracy. Combined with the characteristics of the GRNN itself, when the spread value tends to 0, its function will tend to smooth out, making the training prediction results closer to the test value.

In contrast, the prediction results for new inputs (test set data) that have not been encountered will worsen, a phenomenon known as overlearning [43]. Too large a spread value will make the function more undulating, making it difficult to adapt to test and training values with a is not significant. Therefore, an appropriate spread value for the GRNN needs to consider both the dependent variable of the training samples and the distance between different training samples and the test input samples to make the network more accurate [43,44].

The above analysis shows that the EO-GRNN has the smallest root mean square error, relative error, and absolute error. They all reach a high standard, ensuring prediction accuracy while having credibility. In general, the accuracy of the intelligent optimization algorithm is high. However, the PSO-GRNN and the FOA-GRNN are prone to fall into local optimal solutions, making it difficult to guarantee the accuracy of the prediction results when the measurements are defective. At the same time, the single GRNN is more challenging to guarantee accuracy.

## 5. Conclusions

The GRNN model was established to predict the sound absorption coefficient for the acoustic structure of the three-layer open-cell aluminum foam posterior air layer. The model was improved by using EO, PSO, and FOA optimization algorithms to optimize the solution of the spread values in the GRNN model, respectively. The model with the highest accuracy was obtained as the EO-GRNN model, whose maximum root mean square error was 0.017. In the relative error (−3%,3%) range, the minimum root mean square error was only 0.005, the average root mean square error was only 0.011, and the average relative error and the minimum relative error were 0.006 and 0.001, respectively. Compared with the PSO-GRNN and the FOA-GRNN, it has a better global optimization effect. For the acoustic structures in this study, the obtained EO-GRNN model can be applied to the accurate prediction of the absorption coefficients for small sample sizes, which provides an accurate model basis for the subsequent design of acoustic structures of different sizes. In the future, prediction models more suitable for unfamiliar data can also be developed to solve the problem of poor model prediction in the face of unknown data.

## Figures and Tables

**Table 1:** Basic parameters of two kinds of open-cell aluminum foam.

	Material Code	Porosity (%)	Pore Size (10^−6^ m)	Thickness (10^−3^ m)	Density (g/cm^3^)
A	A_1_	57.185	78	3	1.156
	A_2_	57.185	78	5	1.156
	A_3_	57.185	78	6	1.156
	A_4_	57.185	78	8	1.156
	A_5_	57.185	78	10	1.156
B	B_1_	62.333	67	3	1.017
	B_2_	62.333	67	5	1.017
	B_3_	62.333	67	6	1.017
	B_4_	62.333	67	8	1.017
	B_5_	62.333	67	10	1.017

**Table 2:** SAC of the 10 kinds of single-layer open-cell aluminum foams.

Sample Number	Sample Code	*α*_500_	*α*_630_	*α*_800_	*α*_100_	*α*_1250_	*α*_1600_	*α*_2000_	$\bar{\alpha }$
1	*A*_1_	0.036	0.036	0.083	0.078	0.070	0.095	0.131	0.076
2	*A*_2_	0.060	0.055	0.087	0.109	0.092	0.118	0.154	0.096
3	*A*_3_	0.067	0.072	0.139	0.121	0.127	0.179	0.250	0.136
4	*A*_4_	0.071	0.068	0.106	0.132	0.151	0.211	0.307	0.149
5	*A*_5_	0.077	0.077	0.156	0.166	0.206	0.299	0.432	0.202
6	*B*_1_	0.052	0.043	0.060	0.086	0.079	0.114	0.165	0.086
7	*B*_2_	0.063	0.061	0.083	0.106	0.103	0.143	0.198	0.108
8	*B*_3_	0.061	0.052	0.109	0.120	0.121	0.163	0.228	0.122
9	*B*_4_	0.073	0.060	0.121	0.146	0.169	0.242	0.356	0.167
10	*B*_5_	0.072	0.064	0.146	0.167	0.203	0.298	0.439	0.198

**Table 3:** SAC of the 16 kinds of composite open-cell aluminum foam structures.

Sample Number	Sample Code	*α*_500_	*α*_630_	*α*_800_	*α*_100_	*α*_1250_	*α*_1600_	*α*_2000_	$\bar{\alpha }$
1	*A*_4_ *L*_20_ *A*_3_ *L*_40_ *A*_4_ *L*_10_	0.942	0.924	0.825	0.744	0.677	0.709	0.690	0.787
2	*A*_5_ *L*_30_ *A*_3_ *L*_30_ *A*_1_ *L*_20_	0.899	0.944	0.892	0.811	0.815	0.841	0.851	0.865
3	*A*_5_ *L*_40_ *A*_5_ *L*_30_ *B*_2_ *L*_50_	0.961	0.937	0.920	0.947	0.990	0.985	0.936	0.954
4	*A*_5_ *L*_30_ *A*_4_ *L*_40_ *A*_3_ *L*_10_	0.936	0.902	0.818	0.750	0.737	0.754	0.703	0.800
5	*A*_4_ *L*_40_ *A*_5_ *L*_20_ *B*_4_ *L*_10_	0.816	0.776	0.720	0.685	0.715	0.790	0.737	0.748
6	*A*_4_ *L*_40_ *A*_5_ *L*_50_ *A*_4_ *L*_0_	0.889	0.859	0.774	0.720	0.726	0.765	0.680	0.773
7	*A*_5_ *L*_20_ *A*_2_ *L*_20_ *A*_3_ *L*_20_	0.850	0.952	0.965	0.906	0.832	0.827	0.859	0.884
8	*A*_5_ *L*_30_ *B*_4_ *L*_30_ *B*_5_ *L*_30_	0.850	0.797	0.777	0.767	0.830	0.872	0.885	0.825
9	*B*_4_ *L*_20_ *B*_5_ *L*_30_ *A*_4_ *L*_30_	0.954	0.966	0.926	0.909	0.890	0.780	0.636	0.866
10	*B*_4_ *L*_30_ *A*_5_ *L*_30_ *A*_5_ *L*_20_	0.867	0.832	0.788	0.771	0.803	0.812	0.841	0.816
11	*B*_4_ *L*_40_ *B*_5_ *L*_30_ *A*_1_ *L*_20_	0.931	0.926	0.888	0.847	0.863	0.889	0.850	0.885
12	*B*_5_ *L*_30_ *A*_5_ *L*_30_ *A*_5_ *L*_10_	0.887	0.821	0.765	0.841	0.756	0.760	0.761	0.799
13	*B*_5_ *L*_30_ *A*_2_ *L*_20_ *B*_2_ *L*_20_	0.841	0.862	0.816	0.858	0.807	0.812	0.872	0.838
14	*A*_5_ *L*_30_ *A*_5_ *L*_30_ *A*_4_ *L*_40_	0.939	0.942	0.921	0.926	0.915	0.918	0.842	0.915
15	*A*_2_ *L*_40_ *B*_2_ *L*_50_ *B*_4_ *L*_30_	0.817	0.865	0.719	0.975	0.629	0.809	0.669	0.783
16	*A*_5_ *L*_20_ *B*_5_ *L*_30_ *A*_5_ *L*_10_	0.864	0.879	0.817	0.877	0.714	0.729	0.661	0.792

**Table 4:** Spread values for each model.

Models	Spread Value	Max RMSE	Min RMSE	Ave RMSE
GRNN	2.4	0.120	0.035	0.072
EO-GRNN	0.7	0.017	0.005	0.011
PSO-GRNN	2.2	0.123	0.033	0.072
FOA-GRNN	2.2	0.123	0.033	0.072

## Data Availability

The data presented in this study are available on request from the corresponding author. The data are not publicly available due to restrictions privacy.
